# Reduced Expression of Nogo-A Leads to Motivational Deficits in Rats

**DOI:** 10.3389/fnbeh.2014.00010

**Published:** 2014-01-22

**Authors:** Thomas Enkel, Stefan M. Berger, Kai Schönig, Björn Tews, Dusan Bartsch

**Affiliations:** ^1^Department of Molecular Biology, Central Institute of Mental Health, Medical Faculty Mannheim, University of Heidelberg, Mannheim, Germany; ^2^Schaller Research Group, Division of Molecular Mechanisms of Tumor Invasion, University of Heidelberg and German Cancer Research Center, Heidelberg, Germany

**Keywords:** Nogo-A, reward sensitivity, anhedonia, motivation, avolition, schizophrenia, dopamine, serotonin

## Abstract

Nogo-A is an important neurite growth-regulatory protein in the adult and developing nervous system. Mice lacking Nogo-A, or rats with neuronal Nogo-A deficiency, exhibit behavioral abnormalities such as impaired short-term memory, decreased pre-pulse inhibition, and behavioral inflexibility. In the current study, we extended the behavioral profile of the Nogo-A deficient rat line with respect to reward sensitivity and motivation, and determined the concentrations of the monoamines dopamine and serotonin in the prefrontal cortex (PFC), dorsal striatum (dSTR), and nucleus accumbens (NAcc). Using a limited access consumption task, we found similar intake of a sweet condensed milk solution following *ad libitum* or restricted feeding in wild-type and Nogo-A deficient rats, indicating normal reward sensitivity and translation of hunger into feeding behavior. When tested for motivation in a spontaneous progressive ratio task, Nogo-A deficient rats exhibited lower break points and tended to have lower “highest completed ratios.” Further, under extinction conditions responding ceased substantially earlier in these rats. Finally, in the PFC we found increased tissue levels of serotonin, while dopamine was unaltered. Dopamine and serotonin levels were also unaltered in the dSTR and the NAcc. In summary, these results suggest a role for Nogo-A regulated processes in motivated behavior and related neurochemistry. The behavioral pattern observed resembles aspects of the negative symptomatology of schizophrenia.

## Introduction

Nogo-A is an important neurite growth-regulatory protein in the developing and adult nervous system (Schwab, 2010). While research originally focused on oligodendrocytic Nogo-A and its role in injury and repair of fiber tracts in the CNS, the fact that Nogo-A was found to be present also in neurons (Huber et al., 2002) has risen interest in its involvement in the generation of general behavior, as well. Indeed, in a wide-ranging analysis, Willi et al. (2009, 2010) could demonstrate behavioral alterations in Nogo-A knockout (Nogo-A^−/−^) mice.

Recently, Nogo-A deficiency could be established in the rat species by using a transgenic, constitutively expressed artificial microRNA leading to a 50% reduction of Nogo-A levels in neurons (Tews et al., 2013). Similar to Nogo-A^−/−^ mice, these Nogo-A deficient rats exhibited a variety of behavioral deficits, such as reduced pre-pulse inhibition of the acoustic startle response, behavioral inflexibility, and impairments in short-term memory. In addition, pronounced alterations in social behavior were found. Conducting basic research or preclinical studies in rats offer the advantage that, for example, they more readily learn difficult cognitive behavioral tasks and exhibit more complex social behaviors than mice (Poole and Fish, 1975; McNamara et al., 1996; Costantini and D’Amato, 2006; Cressant et al., 2007). Further, the rat Nogo-A knockdown model uses the well-characterized Sprague Dawley outbred strain and therefore offers increased translational value compared to inbred mice, which is particularly important when evaluating a possible role of neuronal growth regulation in psychiatric disorders (Tews et al., 2013). This latter point is of interest, as the behavioral and structural phenotypes of Nogo-A^−/−^ mice and Nogo-A deficient rats make them potential tools to investigate the pathology of schizophrenia (SCZ; Kristofikova et al., 2013; Willi and Schwab, 2013).

Schizophrenia is a common and debilitating psychiatric disorder and believed to result from neurodevelopmental disturbances (Keshavan et al., 2008; Tandon et al., 2008; Lewis and Sweet, 2009). Interestingly, neuronal Nogo-A is highly expressed particularly during early neuronal development and down regulated later during adulthood in most regions, except the hippocampus, suggesting an important role in neuronal network formation (Huber et al., 2002; Kempf and Schwab, 2013; Mironova and Giger, 2013). In the current study, we aimed to investigate the consequences of Nogo-A deficiency with respect to two important aspects of the negative spectrum of SCZ symptoms, which have not yet been explored in rats nor in mice: *avolition*, a decrease in the motivation to take action and pursue goals, and *anhedonia*, the reduced ability to experience positive affect through reward (Tandon et al., 2009). The negative symptoms of SCZ have been particularly linked to genetic liability and neurodevelopmental disturbances (Dominguez et al., 2010). Further, it has been described before that interference with neuronal development by lesioning the neonatal brain can affect reward sensitivity (Le Pen et al., 2002) or motivated behavior (Schneider and Koch, 2005).

Motivational states in rats can be made accessible to quantification by the use of operant progressive ratio schedules introduced by Hodos and colleagues (Hodos, 1961; Hodos and Kalman, 1963). In this test, subjects need to exhibit progressively increasing effort (more lever pressing) to gain a stable amount of reward; the operant demand at which reward-related responding ceases is termed the “break point” and can serve as an index for reinforcer efficacy or a rat’s motivational state (Barr and Phillips, 1998; Reilly, 1999; Mobini et al., 2000). In the Nogo-A deficient rat, we employed the spontaneous progressive ratio test (PR-Test) and additionally assessed operant responding under extinction conditions, i.e., when rewards were completely omitted. Reward sensitivity was investigated in a well-validated limited access consumption task for sweet rewards (Enkel et al., 2010; Schneider et al., 2010). Finally, to relate behavioral findings to underlying neurochemistry, we analyzed dopamine and serotonin (5-HT) content in brain regions associated with reward processing, namely nucleus accumbens (NAcc), dorsal striatum (dSTR), and prefrontal cortex (PFC).

## Materials and Methods

All experiments in this study were performed in accordance with national and international ethical guidelines, conducted in compliance with the German Animal Welfare Act and approved by the local authorities (Regierungspräsidium Karlsruhe, Germany).

### Subjects

Male, heterozygous Nogo-A deficient rats (Nogo-A KD rats) of the previously characterized and described transgenic line SD-Tg(CAG-RNAi:Nogo-A,EGFP)2ZI (Tews et al., 2013; L2 rats) and wild-type littermates (WT rats) were bred at the animal facilities of the Central Institute of Mental Health, Mannheim. At the beginning of the study they were 8 months old. They were housed in groups of three to four animals per cage under controlled conditions [22°C, 12 h light-dark cycle (lights-on at 7 a.m.), constant humidity]. Throughout the experiments, water was available *ad libitum* and food was restricted to 20 g/rat/day. Each rats’ bodyweight was controlled continuously not to fall below 90% of free-feeding weight. Experiments were performed during the light phase between 09:00 and 12:00. Before the start of behavioral assessments, all rats were handled for 5 min daily on five consecutive days. For behavioral analysis, 18 rats were initially used but 1 rat from the WT group consumed neither the milk used as reward in the experiments nor any other food outside its home cage and it was therefore excluded from the study, resulting in the following group sizes: Nogo-A KD: *n* = 9; WT: *n* = 8. Group sizes in neurochemical experiments were: Nogo-A KD: *n* = 15; WT: *n* = 9; this cohort consisted of randomly chosen animals from the current study and age-matched animals used in a previous study (Tews et al., 2013).

### Consumption test

Prior to the consumption test sessions, rats were given free access to the reward [a 25% solution of sweetened condensed milk (SCM), Milchmaedchen, Nestle Germany] in their home cage to reduce any neophagia. On the day preceding testing, rats were separated in small cages (type 3) for 1 h. Testing for SCM intake took place 24 h later in these cages. After 5 min of habituation, rats had access to a drinking bottle containing the SCM solution for 15 min. The amount of liquid consumed was recorded for each rat by weighing the bottle before and after 5 and 15 min of drinking time. Consume was calculated in relation to the individual body weight. The test was conducted twice, while rats were under the restricted feeding schedule (i.e., in hungry rats) and following overnight *ad libitum* feeding prior to the consumption test (i.e., in non-hungry rats).

### Operant conditioning procedures

All operant schedules were carried out in four identical rat operant training chambers (MedAssociates, Vermont, USA) controlled by a computer running MedPC-IV software and custom-made MedStat Notation code. During sessions, a fan provided constant background noise; sessions always started with illumination of the houselight, which remained on until the session was finished. On the first 2 days, rats received 30 min session of magazine training (levers retracted) in which the dispenser used to deliver 60 μl SCM was manually operated 10–15 times; at the end of the second session all rats reliably drank from the food trough. Magazine training was followed by acquisition of lever pressing under a continuous reinforcement schedule (fixed ratio 1). Each trial started with extension of the response lever. Pressing the lever resulted in its retraction and delivery of 60 μl of SCM; once a rat entered the food trough to consume the reward, the lever was extended for the next trial. Training continued with the so-called “lever access training,” in which rats were required to nose poke into a newly installed response device on the opposite wall of the lever to initiate lever extension. All training sessions ended after 30 min or when 60 rewards had been earned (whatever occurred first). Once all rats showed stable trial initiation and responding, the PR-Test was performed. In a single session (fixed to 30 min duration) the operant demand was progressively increased such that the number of lever presses required to receive a reward was raised by one following each reward delivery (i.e., a dwell 1-step 1 sequence). Following measures were taken: (1) the ratio in which a first inactivity phase of 180 s occurred; this ratio was termed the “break point,” (2) the highest completed ratio achieved within the 30-min session, (3) the latencies to respond on the lever after insertion (response latency), and (4) to consume the reward after delivery (consume latency). Note that after a break point had occurred, a rat was allowed to continue to press the lever for the whole 30 min duration of the session (i.e., the “highest completed ratio” achieved could be higher than or identical to the break point). One day after the PR-Test, lever pressing was tested in a single session under extinction conditions, i.e., all lever presses were unrewarded. To control for activity changes during this session an additional lever with no programed consequences was inserted into the chamber and the numbers of lever presses on both levers were recorded. Both levers were present throughout the session and therefore no nose poking was required.

### Neurochemistry

After decapitation under carbon dioxide anesthesia brains were removed, immediately frozen in isopentane, wrapped in aluminum foil, and stored at −80°. Using a cryotome (Leica, Nußloch, Germany), 120 μm thick frontal sections were cut and brain tissue was dissected from the PFC (from +4.7 to +3.0 mm from Bregma; including medial and orbitofrontal parts), dSTR (from +2.2 to −1.7 mm from Bregma; caudate putamen), and NAcc (from +2.7 to +0.8 mm from Bregma, including NAcc core and shell subregions) using punching needles; respective brain regions were identified according to the Rat Brain Atlas by Paxinos and Watson (2007). Frozen tissue samples were collected in polypropylene tubes and stored at −80° until further processing.

For HPLC analysis samples were thawed, weighed, and immediately homogenized in an extraction solution (0.1 M perchloric acid, 1 mM EDTA) using a tissue homogenizer Mixer Mill (Qiagen, Hilden, Germany). Yielded solutions were subsequently centrifuged at 15000 × *g* for 10 min at 4°C. Cleared supernatants were analyzed by a HPLC system consisting of a Triathlon autosampler (Spark Holland B.V., Emmen, Netherlands), a Shimadzu LC-10 AD pump (Shimadzu Corporation, Kyoto, Japan), a 150 mm × 2.1 mm C18-Reprosil-AQ reverse phase column (3 μm particle size; Dr. Maisch HPLC GmbH, Ammerbuch, Germany) and a Decade II electrochemical detector (Antec Leyden, Zoeterwoude, Netherlands). The mobile phase was 50 mM sodium citrate, 2.4 mM sodium octyl sulfate, 0.1 mM EDTA, 10 mM NaCl, and 22% methanol at pH 4.0. The temperature applied on the system was 37°C. Tissue concentrations were determined by normalizing the quantified amounts of respective neurotransmitters and their metabolites to the corresponding weight of the individual tissue sample.

### Statistics

Sweetened condensed milk intake was analyzed using a three-way repeated measure ANOVA with factors drinking time (5/15 min) × feeding status (restricted/*ad libitum*) × genotype (Nogo-A/WT) and *post hoc* paired *t*-Tests (two-tailed) where appropriate. For operant behavior, lever press activity during training sessions was analyzed as number of trials per minute due to varying session lengths; for the PR-Test, data were analyzed as completed ratios (for example, a ratio of “12” required 12 lever presses to be completed); under extinction, the exact number of lever presses made was taken for analysis. Behavioral parameters and neurotransmitter content were compared using two-tailed Student’s *t*-test or, in cases of unequal variances, Welch’s *t*-Test. Calculations were done with SPSS statistical software (Version 21, IBM Corporation). In all cases *p*-values of 5% or lower (*p* ≤ 0.05) were accepted as statistically significant.

## Results

### Reward sensitivity

**Figure 1:**
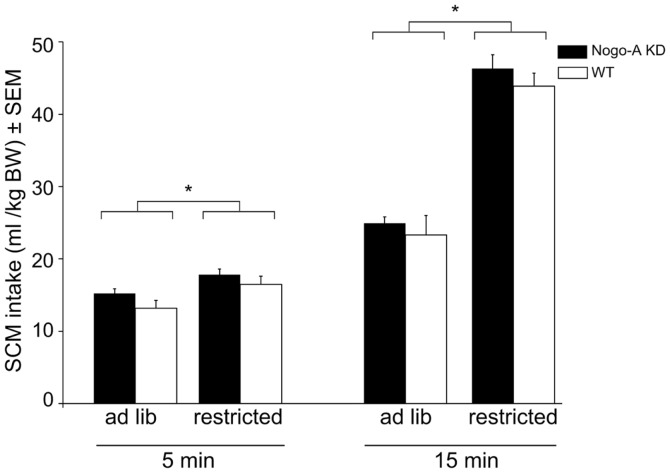
**Reward sensitivity in Nogo-A deficient rats**. There were no differences in SCM intake between Nogo-A KD and WT rats under any feeding condition or at any time point. Restricted feeding prior to the consumption test resulted in higher SCM intake after 5 min (*p* < 0.001) and 15 min (*p* < 0.001). Data are expressed as means ± SEM. WT: *n* = 8, Nogo-A KD: *n* = 9. Asterisks (*) indicate statistically significant differences.

No significant differences were found between the groups of rats regarding their bodyweight on the day before the first consumption test (Nogo-A KD: 632g ± 12; WT:648 g ± 16; *t*_15_ = 0.79, *p* = 0.45). Figure 1 shows the amount of SCM consumed after 5 or 15 min access to the drinking bottle under each feeding status (restricted/*ad libitum*). There was no main effect of the factor genotype (*F*_1,15_ = 0.85, *p* = 0.372) and none of the other factors (feeding state or drinking time) interacted with genotype (all *p* > 0.660), indicating that Nogo-A KD rats consumed similar amounts compared to WT controls under each condition. Significant main effects were found for feeding state (*F*_1,15_ = 158.78, *p* < 0.001) and drinking time (*F*_1,15_ = 457.37, *p* < 0.001), and these two factors interacted significantly (*F*_1,15_ = 113.71, *p* < 0.001); further inspection of the data indicated that, after restricted feeding rats consumed more total SCM in 15 min (*t*_16_ = 12.91, *p* < 0.001) and they also consumed more SCM during the first 5 min of drinking time following restricted compared to *ad libitum* feeding (*t*_16_ = 4.87, *p* < 0.001).

### Motivation

**Figure 2:**
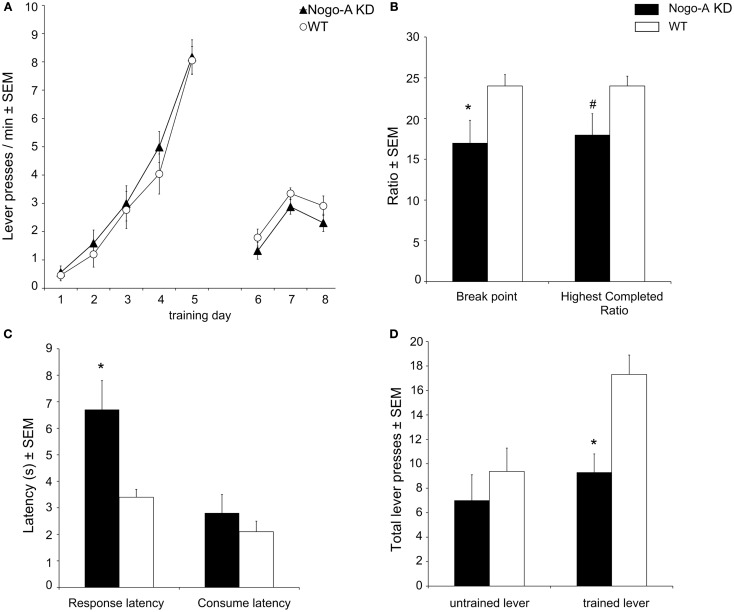
**Motivational behavior in Nogo-A deficient rats**. **(A)** Nogo-A KD rats acquired operant responding similar to WT rats (days 1–5); additionally, there was no difference in baseline performance before the PR-Test (during days 6–8, when nose poking was required to get access to the lever). **(B)** In the PR-Test, Nogo-A KD rats had a significantly lower break point (*p* = 0.049) and tended to have a lower “highest completed ratio” (*p* = 0.088). **(C)** Response latency was increased in Nogo-A KD rats (*p* = 0.014) while the latency to consume the SCM was unaltered (*p* = 0.398). **(D)** Performance under extinction conditions. Nogo-A KD rats made significantly less lever presses than WT rats on the trained lever (*p* = 0.003). There was no difference for the untrained lever (*p* = 0.419). Data are expressed as means ± SEM. WT: *n* = 8, Nogo-A KD: *n* = 9. Asterisks (*) indicate statistically significant differences, the number sign (#) indicates a trend.

Acquisition of operant responding during the initial learning stage (days 1–5), and the performance during the “lever access” stage (days 6–8; nose poking required to access lever) was similar in both strains (Figure 2A). Explicitly, a planned comparison of the number of initiated trials during the last training session before the PR-Test (Figure 2A, day 8) confirmed that there was no statistically relevant difference between Nogo-A KD and WT rats (Nogo-A KD: 2.3 ± 0.3 trials/min, WT: 2.9 ± 0.2 trials/min; *t*_15_ = 1.56, *p* = 0.139). During the PR-Test (Figure 2B), Nogo-A KD rats exhibited a significantly lower break point (Nogo-A KD: 17 ± 2.8, WT: 24 ± 1.4; *t*_11.87_ = 2.19, *p* = 0.049) and, additionally, a tendency for a decreased “highest completed ratio” was found in Nogo-A KD rats (Nogo-A KD: 18 ± 2.6, WT: 24 ± 1.2; *t*_11.22_ = 1.87, *p* = 0.088). Further, response latencies were significantly increased in Nogo-A KD rats compared to WTs (Nogo-A KD: 6.7s ± 1.1, WT: 3.4 s ± 0.3; *t*_8.94_ = 3.04, *p* = 0.014), but consumption latencies were similar between groups (Nogo-A KD: 2.8 s ± 0.7, WT: 2.1 s ± 0.4; *t*_11.78_ = 0.88, *p* = 0.398; Figure 2C). Under the extinction schedule, i.e., when lever pressing was no longer rewarded, responding ceased earlier in Nogo-A KD rats than in WT rats (Figure 2D). While this was apparent for the previously rewarded lever (Nogo-A KD: 9.3 ± 1.5, WT: 17.3 ± 1.6; *t*_15_ = 3.59, *p* = 0.003), this was not the case for the alternatively available new lever (Nogo-A KD: 7.0 ± 2.1, WT: 9.4 ± 1.9; *t*_15_ = 0.83, *p* = 0.419) on which rats from both groups made less responses compared to the previously rewarded lever.

### Neurochemistry

Concentrations of monoamine neurotransmitters and their metabolites in tissue homogenates from PFC, dSTR, and NAcc of Nogo-A KD and WT rats are shown in Table 1. We found a highly significant 25% increase in 5-HT tissue levels in the PFC (WT:1.37 ± 0.07 pmol/mg, Nogo-A: 1.71 ± 0.08 pmol/mg; *t*_22_ = 2.895, *p* = 0.008) while the metabolite 5-Hydroxyindoleacetic acid (5-HIAA) was not altered. No further significant alterations of DA or its metabolites 3,4-dihydroxyphenylacetic acid (DOPAC) or homovanillic acid (HVA) or of 5-HT or its metabolite 5-HIAA were detected in any other brain region measured.

**Table 1:** **Brain tissue concentrations of monoamine neurotransmitters and their metabolites as well as monoamine turnover rates in WT and Nogo-A KD rats**.

Region	Group	DA (pmol/mg)	DOPAC (pmol/mg)	HVA (pmol/mg)	DOPAC/DA	HVA/DA	5-HT (pmol/mg)	5-HIAA (pmol/mg)	5-HIAA/5-HT
PFC	WT	0.20 ± 0.03	0.15 ± 0.01	0.19 ± 0.03	0.81 ± 0.09	1.12 ± 0.35	1.37 ± 0.07	1.68 ± 0.27	1.27 ± 0.25
	Nogo-A	0.17 ± 0.02	0.13 ± 0.01	0.23 ± 0.02	1.25 ± 0.33	2.03 ± 0.50	1.72 ± 0.08**	1.63 ± 0.10	0.96 ± 0.06
dSTR	WT	45.88 ± 4.34	10.23 ± 1.04	2.50 ± 0.28	0.24 ± 0.03	0.06 ± 0.01	1.51 ± 0.21	2.40 ± 0.23	1.66 ± 0.11
	Nogo-A	43.27 ± 2.16	12.01 ± 1.16	2.83 ± 0.39	0.28 ± 0.072	0.06 ± 0.01	1.40 ± 0.11	2.34 ± 0.19	1.69 ± 0.07
NAcc	WT	15.36 ± 2.23	6.28 ± 1.21	1.56 ± 0.36	0.41 ± 0.06	0.10 ± 0.02	1.49 ± 0.26	2.03 ± 0.31	1.44 ± 0.21
	Nogo-A	14.46 ± 1.66	5.68 ± 0.56	1.37 ± 0.15	0.43 ± 0.04	0.10 ± 0.01	1.76 ± 0.21	2.25 ± 0.14	1.52 ± 0.20

*5-HT levels were significantly increased in the PFC*.

Data are expressed as means ± SEM. **p = 0.008; WT: *n* = 9, Nogo-A: *n* = 15

## Discussion

Deficiency of the Nogo-A protein, which is highly expressed during neurodevelopment (Huber et al., 2002), has been associated with a variety of behavioral abnormalities in adult rats (Tews et al., 2013). Here, we extended the behavioral profile of Nogo-A deficient rats with respect to reward sensitivity and motivation.

Consumption of a palatable SCM solution was taken as an indicator for reward sensitivity. This test has been shown to be sensitive to detect anhedonic states, for example, due to blockade of the opioid system (Schneider et al., 2010) or due to stress in an animal model of depression (Enkel et al., 2010). Intake after *ad libitum* pre-feeding with lab chow assumingly reflects mainly the hedonic component of the reward, whereas intake after restricted feeding has an added component of hunger; indeed, when rats were hungry SCM intake was higher and more rapid. Nevertheless, under both conditions Nogo-A KD rats consumed similar amounts of SCM compared to WT and also the time pattern of consumption was similar, implying normal reward sensitivity and translation of hunger into feeding behavior. Our data therefore show that reduced levels of Nogo-A do not affect sensitivity to food rewards. Of course, anhedonia in other domains, e.g., for social reward, cannot be excluded.

Some studies interfering with neurodevelopmental processes have reported unaltered reward sensitivity, but found motivational deficits (Schneider and Koch, 2005). Correspondingly, Nogo-A KD rats exhibited pronounced motivational deficits under a progressive ratio schedule of operant responding. They had a lower break point, indicating that in these rats a “drop” in their motivation to engage in effortful lever pressing occurred earlier, i.e., at lower operant demands. Although some Nogo-A KD rats continued to press the lever after the break point had occurred, they were not willing to invest similar amounts of effort to obtain as much rewards as WT rats, as suggested by the tendency for a decreased “highest completed ratio.” Importantly, inspection of the time courses of responding verified that the 30-min session duration did not pose time constraints on the animals; rather, animals from both groups were inactive during the last minutes of the session. After initiating a trial by nose poking, Nogo-A KD rats were slower to actually begin lever pressing but not to consume the reward once achieved, supporting the interpretation of a motivational deficit but normal reward sensitivity. Our results are not likely to be confounded by impaired learning, since WT and Nogo-A KD rats acquired lever pressing similarly and there were no baseline performance differences between groups prior to the PR-Test, or by decreased locomotor activity, since normal explorative behavior has been shown for Nogo-A KD rats (Tews et al., 2013).

From the current study, the functional origin of the motivational deficit cannot be definitely concluded. Our data allow excluding anhedonia as an explanation, given normal SCM consumption and normal consumption latencies in the PR-Test, but instead suggest disturbances in the instrumental phase of motivated behavior. The latter refers to several processes required to translate reward information into goal-directed behavior, such as “wanting,” cost-benefit calculation and response initiation, which are more difficult to disentangle (Salamone and Correa, 2012). The fact that Nogo-A KD rats still initiated trials by nose poking to access the lever and performed lever pressing to a certain extent suggests normal response initiation. Further, this also suggests that the reward was still “wanted” and that a representation of the reward could be used to motivate them to start a new trial. But there are some hints that in Nogo-A KD rats either the representation of reward value itself or its use in cost/benefit calculations could indeed be altered. Interesting in this respect is a comparison of the performance in the PR-Test with the extinction session. In both groups, lever pressing ceased earlier under extinction conditions than in the PR-Test, but this effect was much stronger in Nogo-A KD rats. The main difference between these sessions is that during extinction any lever pressing is solely dependent on a representation of the reward (since no rewards can be physically experienced), while during the PR-Test the action-reward interval increases progressively and provides at least some hedonic experience (some rewards are delivered). Importantly, on the (never rewarded) control lever both groups made comparably few responses excluding overall lower activity in Nogo-A KD rats during extinction as an alternative explanation.

Our study also collected neurochemical data related to motivational processes. Although, our analysis of tissue monoamine levels under baseline conditions does not allow drawing substantial conclusions about direct functional relationships between altered monoaminergic activity and behavior, these data nevertheless provide some indirect information on the processes potentially contributing to the motivational deficit. Dopamine has been particularly linked to “wanting” and cost/benefit calculations (Salamone et al., 2007; Salamone and Correa, 2012) and the fact that we found no alterations in the dopaminergic transmitter system was surprising, but appears to be in line with our above discussed observation that Nogo-A KD rats were still wanting the reward. Of particular interest is therefore the highly significant increase in prefrontal 5-HT levels in Nogo-A KD rats. Increased brain serotonergic tone, due to deletion of the serotonin transporter or chronic 5-HT reuptake blockade, has been shown to decrease motivation for natural rewards in mice (Sanders et al., 2007). Particularly intriguing, increased serotonergic tone in the orbitofrontal areas of the PFC has been specifically associated with impaired ability to use the value of expected outcomes to guide behavior (Nonkes et al., 2010). This suggests that the motivational deficit in Nogo-A KD rats could indeed be a consequence of the observed alterations of 5-HT levels in the PFC. Brain microdialysis experiments monitoring transmitter release in the behaving rat could provide such correlative information. Alternatively, alterations in transmitter systems other than the dopaminergic or serotonergic systems could of course contribute to the motivational deficit. For example, the glutamatergic and GABAergic systems, which could not be measured in this study, have also been linked to motivational processes (Faure et al., 2010).

The findings of this study are of considerable interest given that reward-related disturbances are clinical features of many psychiatric disorders (Brown and Pluck, 2000), e.g., of the negative symptoms of SCZ which have been particularly linked to genetic liability and neurodevelopmental disturbances (Tandon et al., 2009; Dominguez et al., 2010). In our study Nogo-A KD rats did not show deficits in reward sensitivity; although anhedonia – which is defined as the decreased capacity to experience pleasure – has long been considered a symptom in SCZ, more recent research supports the idea that hedonic processing itself is indeed normal in patients but that cognitive deficits instead lead to negatively biased self-report about hedonic experience (Strauss and Gold, 2012). More important in this respect is that the motivational deficit in Nogo-A KD rats resembles avolition, which is defined as a lack in the motivation to pursue goals. Avolition is commonly seen in patients with SCZ and has always been considered a core negative symptom (Tandon et al., 2009; Messinger et al., 2011). Furthermore, an increase in serotonergic neurotransmission is suggested to be present in schizophrenic patients (Ngan et al., 2000; Dean, 2003). Our current results are therefore in line with earlier reports linking the behavioral phenotype of Nogo-A deficient rats (Tews et al., 2013), or of Nogo-A^−/−^ mice (Willi et al., 2010), to a variety of schizophrenic symptoms.

The fact that the rats used in the current study and by Tews et al. (2013) were deficient of Nogo-A in neurons, but not in oligodendrocytes, allows an interesting comparison with earlier work in mice which bear the Nogo-A knockout in both cell types (Willi et al., 2010). Similar to these mice, Nogo-A KD rats exhibit behavioral deficits such as reduced pre-pulse inhibition of the acoustic startle response, behavioral inflexibility, impairments in short-term memory and impairments in management of reference frames (Tews et al., 2013; Petrasek et al., 2014). Additionally, they show the motivational deficits demonstrated in the current study. Given the cell-type specific temporal expression profile of Nogo-A, with high expression of Nogo-A in neurons mainly during early development and high expression in oligodendrocytes mainly in the postnatal nervous system, the behavioral deficits observed in the Nogo-A deficiency rat model and the Nogo-A^−/−^ mouse model appear to be largely the consequences of disturbed brain developmental processes following Nogo-A deficiency particularly in neurons. Indeed, Nogo-A has been shown to be critically involved in early cortical development and neuronal maturation (Mingorance-Le Meur et al., 2007). This is intriguing since SCZ is considered to be a disorder of abnormal neurodevelopment (Lewis and Sweet, 2009). Notably, we found some clear differences between Nogo-A KD rats and Nogo-A^−/−^ mice regarding alterations in neurotransmission: in contrast to rats, Nogo-A^−/−^ mice had reduced tissue levels of DA and its metabolites DOPAC and HVA in the dSTR. Additionally, no reductions in tissue levels of 5-HT were found (Willi et al., 2010). Whether these differences to rats are related to the additional lack of oligodendrocytic Nogo-A in mice is unclear. Yet, since oligodendrocytic Nogo-A is crucial for myelination, which continues postnatally, ongoing perturbations in neuronal development might lead to the differential outcome in Nogo-A KD rats and Nogo-A^−/−^ mice. Alternatively, in Nogo-A^−/−^ mice compensatory upregulation of Nogo-B has been reported (Willi et al., 2010), an effect not present in Nogo-A deficient rats (Tews et al., 2013). More research is needed to determine possible differential contributions of neuronal or oligodendrocytic Nogo-A to the behavioral and neurochemical phenotypes observed. The Nogo-A deficiency rat model nevertheless provides a promising tool to complement the Nogo-A^−/−^ mouse model to further elucidate the role of Nogo-A in neuropsychiatric disorders like SCZ.

## Author Contributions

Thomas Enkel, Kai Schönig, Björn Tews, and Dusan Bartsch initiated and designed the study; Thomas Enkel and Stefan M. Berger performed experiments; Thomas Enkel, Stefan M. Berger, Kai Schönig, Björn Tews, and Dusan Bartsch wrote the manuscript.

## Conflict of Interest Statement

The authors declare that the research was conducted in the absence of any commercial or financial relationships that could be construed as a potential conflict of interest.
